# Is the Diagnosis of Skin Diseases Necessary?

**Published:** 1896-09

**Authors:** M. B. Hutchins

**Affiliations:** Clinical Lecturer on Skin Diseases and Syphilis, Atlanta Medical College, Atlanta, Ga.; 311-312 Fitten Building


### IS THE DIAGNOSIS OF SKIN DISEASES NECESSARY?

By M. B. HUTCHINS, M. D.

Clinical Lecturer on Skin Diseases and Syphilis, Atlanta Medical College, Atlanta, Ga.

Many able and eminent practitioners have told me that they knew nothing about skin diseases. It is a pretty well established rule that we are indifferent to work about the doing of which we know nothing, and that the more we know of a subject the more it interests us, and the more appreciated is its importance. The practitioner who does not understand diseases of the skin is inclined to undervalue their importance and to treat them haphazard when he is consulted.

If skin diseases are of no importance a diagnosis is not necessary and the dermatologist "lags superfluous."

But the diagnosis and proper treatment of skin diseases, and those diseases with skin manifestations, is necessary in the majority of cases, and their neglect may lead to a train of consequences disastrous to the patient, and injurious to his physician.

The diagnosis of a skin disease is impossible to the general practitioner in most cases, and not always easy to the dermatologist.

The physician with special training and experience is alone qualified to judge of the importance or unimportance of a skin eruption. The diagnosis of eczema, and the application of zinc ointment, will suffice in very few cases. Eczema alone may embrace every grade of cutaneous inflammation, the stages of the disease requiring as different treatment as entirely dissimilar diseases.

A child may be isolated, and the family subjected to months of inconvenience, under the diagnosis of scarlet fever when the case is only scarlatiniform erythema, or, as I once saw, a kerosene oil dermatitis may be called scarlet fever because the papillæ of the tongue were prominent.

An infant may be treated for an intertrigo, or an eczema intertrigo, and finally go into coma and die, the physician then finding that he has had a case of congenital syphilis.

A poor negro may be called syphilitic when he has a profuse lichen, or keratosis follicularis, pustular eczema, acne cachecticorum, or some other disease deceptive through its grouping and distribution.

A young girl came here from a distant state to be treated for a severe "nasal catarrh." She had syphilis, the cartilaginous part of the nose and portions of the bones being destroyed. Antisyphilitic treatment cured her, and a successful rhinoplasty was done three years later.

Lupus vulgaris, syphilis, epithelioma may be confused, the one with the other, or may co-exist. A diagnosis would seem to be necessary to the application of proper treatment. Impetigo contagiosa or furunculosis or carbuncle have been diagnosed and treated as constitutional diseases, when thorough local and antiseptic treatment, with or without general tonics, were alone required.

An applicant for a position on the police force was sent away as syphilitic when he had an eczema.

Innocent young men have felt the "finger of scorn" though they had only an acne.

A young lady teacher was sent to me to learn if she had "the itch," which would cause the loss of her position. She had a vesicular eczema.

A nurse girl was in constant care of an infant until the mother was told that her nurse had "itch"—scabies—and must go. A cook and nurse had an eczematoid skin with "itch" lesions. She was soaked in "lye" soap and water, followed by quantities of sulphur ointment, and the little children escaped contagion.

A woman from Collins street had a scant, livid papular eruption. It was syphilis, and she was warned of the probable danger to her visitors.

Another came for "cancer" of the rectum. The anus, perineum and labia were nearly destroyed by chancroid.

A man had some brownish patches on his chest. It was tinea versicolor, due to a fungus, and contagious.

A young man with acne happened to mention a sore on his penis. It was a large chancre, and he had the macular eruption.

The examples cited are not exceptional, and are sufficiently numerous to show that there are many skin diseases, upon the proper diagnosis of which depends the welfare, not only of the patient, but often also that of those who come in contact with him.

There is a pleasant side to the question, as well. Many people are rendered miserable by their own, or the physician's opinion, that they are syphilitic. The most agreeable experience I have, is to be able to assure these people that they are not syphilitic, in spite of what they think a positive history and certain symptoms of the disease.

That wrong treatment must follow wrong diagnosis there is abundant evidence. For example, it is only necessary to point to syphilis, or to consider the possibility of treating a case surgically which would yield to medical treatment, or of treating a case of epithelioma or *ingrowing toe-nail* with internal medicines. I believe it is best to treat a cutaneous tuberculosis, where practicable, by excision, and in so far does the treatment resemble that of operable epithelioma, but the operation for epithelioma must be the more thorough and extensive.

A case illustrating the harm of an erroneous diagnosis and "history" came under my observation in a consultation. A young man had had an indefinite lesion wh ch was thought to have been chancre. There was never a skin eruption. A number of years afterward his wife had some eruption on her face. He raked up his old history for the physician, and the lady was straightway saturated with iodide of potassium. When I saw her, her face was disfigured with an extensive iodic eruption which the physician had considered a further evidence of syphilis, continuing the iodide. The only treatment required was stopping the medicine, and the local use of a sedative ointment.

There are ill-defined skin eruptions which scarcely deserve a name. There are skin eruptions, the diagnosis of which is not essential, they are so trivial. There are other skin eruptions, masked by treatment, altered by scratching, or so complex that even experts may differ as to the diagnosis, or a diagnosis is not possible. When a diagnosis is made one should be so certain of its correctness that he would be willing to state his opinion on oath.

Now, in comparison, notice the patients coming before any practitioner, or observe the material at a free clinic, general or special. Are there not many trivial cases; are there not many obscure cases, and are there not many undiagnosable cases? Does this demonstrate that there are no cases necessary to be diagnosed?

Viewed from any standpoint, the more cases we are able to diagnose the more cases are we able to treat properly, and the fewer regrets rest upon our conscience.

As intimated above, a diagnosis is often all that is desired or can be done. The ability to make this diagnosis either enables the physician to lift a weight of dread and suspense from the patient's mind, or to change the horrible insomnia of uncertainty into the deep sleep of resignation to the certain fate which has overtaken him.

*311–312 Fitten Building.*

